# White spot lesion management: Knowledge and practices during fixed orthodontic treatment among Saudi dentist -A cross-sectional study

**DOI:** 10.6026/973206300214492

**Published:** 2025-12-15

**Authors:** Hammad Saleh Alshyai, Muhammad Atif Saleem Agwan, Nada Mohammed Aloufi, Eyad A AlTamimi, Abdullah A Aloyouni, Batool Nasser Alhabib

**Affiliations:** 1 College of Dentistry, Qassim University, Kingdom of Saudi Arabia; 2 Department of Conservative Dental Sciences, College of Dentistry, Qassim University, Kingdom of Saudi Arabia; 3 Department of Orthodontics and Paediatric Dentistry, College of Dentistry, Qassim University, Kingdom of Saudi Arabia; 4 Department of Preventive Dental Sciences, College of Dentistry, King Saud bin Abdulaziz University for Health Sciences, Riyadh, Kingdom of Saudi Arabia

**Keywords:** Knowledge, practice, white spot lesions, years of experience

## Abstract

White spot lesions (WSLs), a consequence of orthodontic treatment, have a detrimental effect on the aesthetic result of orthodontic treatment. Therefore, it is of interest to assess knowledge and practice levels of dentists practicing orthodontics regarding the prevention and treatment of WSLs in Saudi Arabia. Hence, this cross-sectional observational study included 188 orthodontic practitioners in Saudi Arabia with at least one year of experience utilizing structured electronic questionnaire of 19 closed-ended items. Knowledge assessment revealed that 133 (70.7%) had high scores and 55 (29.3%) had intermediate scores while in practice assessment, 154 (81.9%) achieved high scores, while 34 (18.1%) had intermediate scores with most participants having 1-4 years of experience demonstrated high knowledge (63.4%) and practice (76.8%) levels. Thus, we show that the dentist practicing orthodontics showed high knowledge and practice regarding the prevention and treatment of white spot lesions with years of experience were significantly associated with the levels of knowledge.

## Background:

In recent times, orthodontic procedures have become increasingly common and one of the main issues with dental aesthetics is the white, chalky patches that follow treatment also referred to as white spot lesions, or WSLs [1]. White spot lesions (WSLs) can be seen as big decalcified areas, either with or without cavitation in some cases, or they can appear as tiny lines surrounding the brackets. Numerous factors are involved in enamel demineralization, such as improper diet and insufficient oral hygiene, as well as the use of inappropriate adhesive methods [2]. Above all, new retentive areas created by orthodontic appliances such as brackets, ligatures, and arches in particular have the unintended consequence of promoting plaque accumulation [3]. The primary cause of the enamel's deterioration during orthodontic procedure is an increase in the quantity of dental plaque containing cariogenic bacteria. WSLs, or even caries, appear as a result of this demineralization of the tooth surfaces. Nonetheless, conflicting findings about the link between orthodontic treatment and the emergence of dental caries have been reported in the previous literature [4]. The buccal regions of teeth are often the sites of WSLs. These lesions primarily affect the canines and upper lateral incisors of the teeth [5]. The reduction of enamel mineralization has been identified in these regions, which can be characterised clinically as more or less large, porous, rough to the touch, and chalky white or brown areas. This process is linked to different light diffusion in these regions than in normally mineralized enamel [6]. If these enamel lesions are left untreated, they will progress into caries [7]. These demineralization lesions may occasionally be reversible, and salivary proteins that remineralize the enamel surface can partially mask the chalky appearance [8]. However, in the case of orthodontic procedures, these enamel lesions develop gradually, and irreversibly, leading the development of caries [9]. The patient experiences an aesthetic issue after orthodontic treatment due to the WSLs, which has a prevalence ranging from 2 to 96% [10]. There have been reports of a number of preventive methods to reduce the incidence of WSLs, including professional fluoride application (fluoride varnish), frequent daily tooth brushing, and use of fluoridated mouthwash. Additional innovative approaches were also suggested, such as replacing fluoride-containing dentifrices with enzyme dentifrices like amyloglucosidase and glucose oxidase [11]. The demineralization of the enamel surface results in white spot lesions, which are aesthetic issues. WSLs are more likely to develop around orthodontic attachments because orthodontic treatment creates hard-to-clean areas that are more likely to accumulate plaque. Therefore, it is of interest to assess the knowledge and practice levels of dentists practicing orthodontics about the prevention and treatment of WSLs in Saudi Arabia.

## Materials and Methods:

This cross-sectional observational study was performed on dentists practicing orthodontics in Saudi Arabia, using a non-probability convenient sampling technique. The duration of the study was about three months from January 1st 2024 to March 30th 2024. The ethical approval was obtained from Qassim Region Research Ethics Committee with approval number 607/45/7072. A total of 188 dentists practicing orthodontics who had at least one year of experience treating patients with fixed orthodontics, and were willing to participate in this study were included. In contrast, dentists with less than a year of orthodontic practice experience as well as those who did not complete the questionnaire were excluded from the study. A structured electronic questionnaire consisting of 19 closed-ended questions was used to collect data. The questionnaire comprised three sections: the first gathered socio-demographic details about the participants; the second assessed the orthodontists' knowledge of WSL; and the third section investigated their use of clinical preventive measures in practice. The Likert scale was employed in creating the questionnaire, and its validity and reliability were confirmed in previous study [12]. The participants were made aware that their information would be kept private and that their participation was entirely voluntary. The knowledge and practice questions were scored according to the following system: Participants received 4 points for selecting "totally agree," 3 points for "agree," 2 points for "disagree," and 1 point for "totally disagree." Their scores were calculated by adding up the points from all the related questions. As stated by Daniels (1987), a three-level index score system (low, intermediate, and high) was developed for both knowledge and practice in preventing and treating WSLs. This methodology involved assigning 36 points for practice and a total of 40 points for knowledge.

Regarding the knowledge scores, the levels were divided into 3 categories:

Group 1: ≤20 (score for low knowledge).

Group 2: 21-30 (score for intermediate knowledge).

Group 3: 31 -40 (score for high knowledge).

Three groups comprised the final levels in the practice score:

Group 1: ≤18 (score for low practice)

Group 2: 19-27 (score for intermediate practice).

Group 3: 28- 36 (score for high practice).

## Statistical analysis:

The descriptive analysis of the data was analysed using the Social Science Statistical Package (SPSS; Armonk, NY, USA) version 26.0. The qualitative data was documented as frequencies and percentages, and quantitative variables were reported as means and standard deviations. The Mann-Whitney test was utilized to compare the knowledge and practice levels among participants based on gender and postgraduate orthodontic degree. Additionally, the Kruskal-Wallis test was employed to compare knowledge and practices among orthodontists with different years of experience. A chi-square test was used to find the relationship between knowledge and practice assessment scores with respect to gender and years of experience. A p value of less than 0.05 was considered statistically significant.

## Results:

A total of 188 dentists practicing orthodontics were included in this study, of 106(56.4%) were males and 82(43.6%) were females, with the mean age of the participants was 29.32 ± 3.86 years. Approximately 116(61.7%) of the participants held an orthodontic postgraduate degree, while 72(38.3%) did not. The distribution of years of professional experience varied, with 112(59.6%) individuals having 0 to 4 years, 70(37.2%) having 5 to 10 years, and only a small percentage having 11 to 20 years of experience, as depicted in Table 1 (see PDF). In terms of knowledge assessment, none of the participants scored low (≤20), while 55(29.3%) scored at an intermediate level (21 - 30) and 133(70.7%) scored high (31-40). For practice assessment, no one scored low (≤18), 34(18.08%) scored at an intermediate level (19 - 27), and 154(81.92%) scored high (28-36), as illustrated in Figure 1 (see PDF). The knowledge and practice scores of the participants categorized by gender and years of experience revealed that 71(63.4%) and 86(76.8%) participants with 1-4 years of experience predominantly exhibited a high level of both knowledge and practice scores, respectively. Similarly, those with 5-10 years of experience showed a high level of knowledge 56(80.0%) and practice 63(90.0%) scores, with a smaller proportion revealing intermediate scores. Across gender, both males and females showed high levels of both knowledge and practice scores. However, significant differences were observed in knowledge scores across different years of experience (p = 0.040), while an insignificant differences were found between genders in both knowledge (p = 0.334), and practice (p = 0.948) scores, as depicted in Table 2 (see PDF). The participants' responses to knowledge-related questions regarding WSLs in orthodontic practice showed that the most participants agreed that WSLs are a main orthodontic problem, with 101 (53.7%) totally agreeing and 84 (44.7%) agreeing. Regarding prevention, 65 (34.6%) completely agreed and 107 (56.9%) agreed. On treatment, 120 (63.8%) totally agreed and 65 (34.6%) agreed. For reversibility, 75 (39.9%) totally agreed and 82 (43.6%) agreed, while 6 (3.2%) totally disagreed and 25 (13.3%) disagreed. Nearly all, 91 (48.4%) totally agreed and 93 (49.5%) agreed, believed WSLs represent sub-surface enamel demineralization. Concerning affected teeth, 101 (53.7%) agreed and 58 (30.9%) totally agreed that the maxillary lateral incisor and mandibular canine are most affected. Similarly, 105 (55.9%) totally agreed and 76 (40.4%) agreed that longer orthodontic duration increases WSL prevalence. Early lesion development around attachments was supported by 58 (30.9%) totally agreeing and 91 (48.4%) agreeing. On gender differences, 62 (33.0%) totally agreed and 63 (33.5%) agreed that males are more affected. Finally, 55 (29.3%) totally agreed and 103 (54.8%) agreed that posterior segments are the least affected sites, as depicted in Table 3 (see PDF). The participants' responses to practice-related questions regarding orthodontic treatment and the management of WSLs showed that the most participants, 117(62.2%) totally agreed and 68(36.2%) agreed, that patients should be made aware of caries risk during orthodontic treatment. Similarly, 119(63.3%) totally agreed and 67(35.6%) agreed that orthodontists should provide oral health instructions. The recommendation of fluoridated mouthwash was supported by 73(38.8%) totally agreeing and 95(50.5%) agreeing. Regular check-ups for caries and WSLs were endorsed by 96(51.1%) totally agreeing and 87(46.3%) agreeing. Reminding patients of oral hygiene and prescribing home care products was considered effective by 98(52.1%) totally agreeing and 81(43.1%) agreeing, while the same proportions, 98(52.1%) and 81(43.1%), supported professional fluoride application as an ideal method. Microabrasion was accepted by 70(37.2%) totally agreeing and 94(50.0%) agreeing, and bleaching by 81(43.1%) totally agreeing and 85(45.2%) agreeing. Finally, resin infiltration was regarded as a minimally invasive treatment by 111(59.0%) totally agreeing and 73(38.8%) agreeing, as depicted in Table 4 (see PDF). A comparison of knowledge and practice scores among participants with respect to sociodemographic features revealed that in terms of knowledge, an insignificant differences were observed between both genders (p = 0.252) and presence of postgraduate orthodontic degree (p = 0.654). However, a significant difference was found in knowledge scores across different years of experience (p = 0.001), with participants having 16-20 years of experience demonstrating the highest mean score (36.0 ± 4.58). Similarly, an insignificant differences were noted between both genders (p = 0.695) or possession of a postgraduate orthodontic degree (p = 0.096), with respect to practice scores. While there were no significant differences in practice scores across different years of experience (p = 0.156), participants with 5 -10 years of experience indicated the maximum mean practice score (31.57 ± 2.42), as depicted in Table 5 (see PDF).

## Discussion:

Orthodontic treatment has become more common due to the sharp rise in demand for dental aesthetics. The present study demonstrated the knowledge and practice levels for the prevention of WSLs among dentists practicing orthodontics in Saudi Arabia. A cross-sectional study of 60 orthodontic specialists and dentists assessed knowledge and practices regarding WSLs prevention and treatment. Most participants (78.3%) had intermediate knowledge, while 66.7% showed high practice scores. Male orthodontists and those without postgraduate degrees scored significantly lower than females and postgraduates (p = 0.001). Overall, participants demonstrated adequate knowledge and practices concerning WSLs [12]. The present study partially concurred with the above-mentioned study and indicated that the majority of participants, 101(53.7%) totally agreed and 84(44.7%) agreed with the statement that WSLs is a major problem in orthodontics. Most of the participants had high knowledge and practice scores. However, significant differences were observed in knowledge scores across different years of experience (p = 0.040), while an insignificant differences were observed between genders in both knowledge (p = 0.334) and practice (p = 0.948) scores. According to one study, a majority of the participants (80%) concurred that resin infiltration is a minimally invasive restorative therapy suitable for treating WSLs. Half of the participants disagreed that micro-abrasion should be considered one of the treatment possibilities for WSLs [13]. Additionally, micro-abrasion and resin-infiltration therapies can reduce the white appearance of WSLs [14]; it lessens the disparity between the WSLs and the remaining enamel surface by its effect, which is inconsistent with a systematic review, found no conclusive evidence in favour of the use of bleaching as a successful treatment for post-orthodontic WSLs [15]. The present study was inconsistent with the above-cited studies and indicated that 98(52.1%) of the participants totally agreed, and 81 (43.1%) agreed that professional fluoride application is the best course of action for treating WSLs. About 94(50.0%) agreed that microabrasion is also considered a viable treatment method for WSLs. Furthermore, 81(43.1%) participants totally agreed, and 85 (45.2%) agreed that bleaching is one of the approaches used in WSLs treatment. Additionally, 111(59.0%) participants totally agreed that resin infiltration is a minimally invasive restorative therapy for post-orthodontic WSLs. Another cross-sectional study of 109 Iranian orthodontists (40.6% males, 59.4% females) assessed practices for WSLs prevention and treatment. Most (94.4%) used fluoride products, with toothpaste being the most common (77%), while professional fluoride therapy was least prescribed. Fluoride mouthwashes and toothbrushes were also frequently recommended (34%). Female orthodontists scored significantly higher than males (p = 0.001). Practices improved with age (p = 0.001), but not with years of experience (p = 0.230) [16]. In contrast to the cited study, the present study of 188 participants (56.4% males, 43.6% females) found that most considered fluoridated mouthwash essential alongside toothpaste for preventing WSLs. No significant gender differences were observed in knowledge scores (p = 0.252), but a significant difference was noted across years of experience (p = 0.001), with the highest scores seen among those with 16-20 years of experience (36.0 ± 4.58). According to a recent study conducted in the USA, the most commonly mentioned recommendation against WSLs therapy following multi-bracket therapy was fluoridation, followed by resin infiltration [17]. It was also observed in another study that the topical material has demonstrated unsatisfactory results in reversing WSLs [18]. In addition to stopping the enamel lesions, resin infiltration is a less invasive way to enhance the cosmetic results of multi-bracket therapy [19, 20]. The present study revealed that a significant majority, with 98(52.1%) totally agreeing and 81(43.1%) agreeing, preferred professional fluoride application is one of the most ideal, practical ways to defeat WSLs. Around 111(59.0%) participants totally agreed, and 73(38.8%) agreed that resin infiltration is a minimally invasive restorative approach for post-orthodontic WSLs. This study's scope is limited because it didn't explore how often WSLs occur among patients in the study population. As a result, we couldn't establish a connection between orthodontists' expertise and awareness of WSLs and how often their patients develop WSLs. Additionally, the study didn't investigate the viewpoints of patients regarding the prevalence of WSLs.

## Conclusion:

The dentist practicing orthodontics showed high knowledge and practice regarding the prevention and treatment of white spot lesions. Moreover, years of experience were significantly associated with the levels of knowledge regarding the prevention and treatment of white spot lesions. However, gender and holding a postgraduate orthodontic degree showed no significant association with knowledge and practices.

## Author contributions:

## Funding:

This research received no external funding.

## Institutional review board statement:

The study was conducted in accordance approved by Qassim Region Research Ethics Committee with reference number 607/45/7072.

## Informed consent statement:

Informed consent was obtained from all subjects involved in the study.

## Data availability statement:

The data that support the findings of this study are available from the corresponding author upon reasonable request.
